# Dysfunction of microstructure and metabolism in corpus callosum in juvenile schizophrenia

**DOI:** 10.1192/j.eurpsy.2023.1284

**Published:** 2023-07-19

**Authors:** M. Ublinskiy, A. Manzhurtsev, T. Akhadov, I. Lebedeva

**Affiliations:** 1 Radiology, Clinical And Research Institute Of Emergency Pediatric Surgery And Trauma; 2 Psychiatry, Psychiatry- National Mental Health Research Centre, Moscow, Russian Federation

## Abstract

**Introduction:**

The corpus callosum (CC) is one of the important structures responsible for communication between the brain hemispheres. Its role is particularly important in cognitive tasks performance, information processing, concentration of attention, in mnestic processes. The corresponding dysfunctions are the major symptoms of schizophrenia, and hence, structural characteristics of CC in schizophrenics are in the focus of attention.

**Objectives:**

The aim of the study was to analyze the microstructural and metabolic features of the corpus callosum in recently onset schizophrenia.

**Methods:**

The study was carried out in 13 men with juvenile endogenous paroxysmal psychosis (disease standing ≤ 5 years after first manifestation) aged 17-27 years (median 22.0±3.1 years). The studies were carried out during unfolding remission or in remission. Control group consisted of 15 mentally healthy young men (18-28 years). MRI and 1H-MRS studies were carried out on Achieva 3T MRI scanner device (Phillips). Diffuse tensor images were obtained in the axial plane using echo-planar pulse sequence. Diffuse gradients were applied in 32 noncolinear vectors. The spectra were recorded by single voxel 1H-MRS. The spectroscopic voxel (2×1×1 cm) was placed in the CC genu region. The PRESS sequence was used (TR/TE=1500/40 msec).

**Results:**

Statistical analysis showed no abnormal diffusion values in the CC splenium in the patients. Significant changes in the parameters were found in the CC genu. The values of ADC and RD increased, while FA coefficient decreased in the CC genu of patients with the initial stage of schizophrenia; PD values were normal. The increase of RD in the presence of unchanged PD indicated a decrease of water diffusion velocity and anisotropy in the direction perpendicular to the axon orientation. A typical 1H-MRS of the CC genu was presented in Figure 1. The results of statistical analysis of metabolite signal intensities in the CC genu of patients and normal subjects were presented in Figure 2. NAA level was reduced significantly in the patients. No appreciable changes in Cho values in the CC genu were detected in the patients vs. normal subjects.

**Image:**

**Figure:**
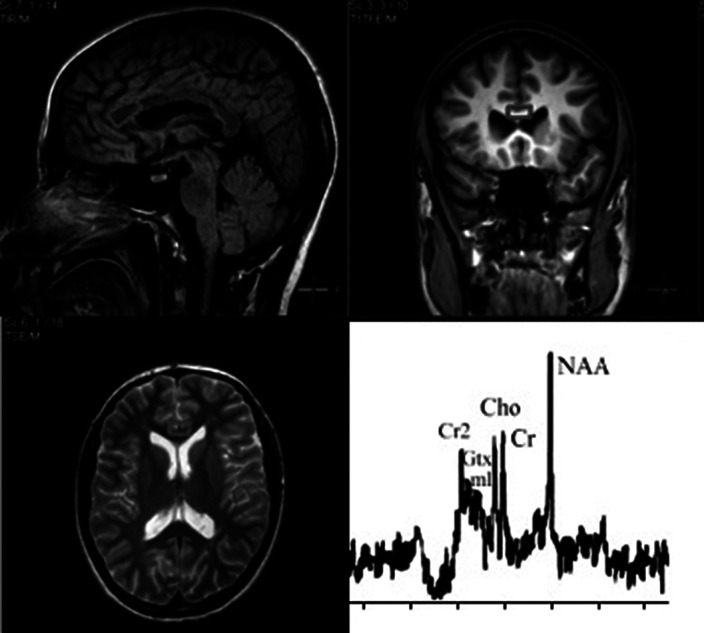

**Image 2:**

**Figure:**
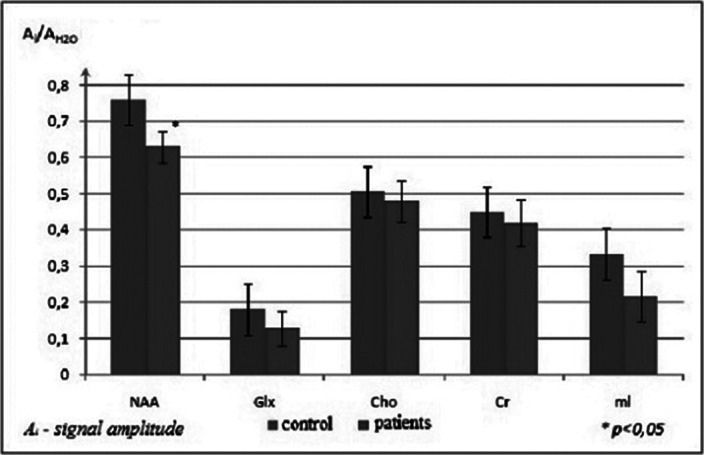

**Conclusions:**

The increase of RD could be caused by several factors: impairment of myelin membranes, axon damage because of impairment of axon cytoskeleton, and changed organization of fibrils. Our results showed that RD increase in patients with early schizophrenia did not conform to active demyelination, which was proven by the normal level of Cho, while axon damage, shown by low level of NAA, did not lead to PD reduction.

The decrease of NAA level detected in our study indicated axonal damage in the CC genu of patients in the early stage of schizophrenia. The increase of RD in the presence of normal Cho level seemed to indicate disorders in the axon cytoskeleton damage, but not active demyelination.

**Disclosure of Interest:**

None Declared

